# Decreased GABA levels of the anterior and posterior cingulate cortex are associated with executive dysfunction in mild cognitive impairment

**DOI:** 10.3389/fnins.2023.1220122

**Published:** 2023-08-11

**Authors:** Xiaona Fu, Mengting Qin, Xiaoming Liu, Lan Cheng, Lan Zhang, Xinli Zhang, Yu Lei, Qidong Zhou, Peng Sun, Liangjie Lin, Ying Su, Jing Wang

**Affiliations:** ^1^Department of Radiology, Union Hospital, Tongji Medical College, Huazhong University of Science and Technology, Wuhan, China; ^2^Hubei Province Key Laboratory of Molecular Imaging, Wuhan, China; ^3^Department of Neurology, Union Hospital, Tongji Medical College, Huazhong University of Science and Technology, Wuhan, China; ^4^Clinical & Technical Solutions, Philips Healthcare, Beijing, China

**Keywords:** mild cognitive impairment, anterior cingulate cortex, posterior cingulate cortex, GABA, executive function, MEGA-PRESS

## Abstract

**Background and purpose:**

Executive function impairment, a slight but noticeable cognitive deficit in mild cognitive impairment (MCI) patients, is influenced by gamma-aminobutyric acid (GABA) levels. Reduced cognitive function is accompanied by thinning of the cerebral cortex, which has higher GABA levels than white matter. However, the relationships among GABA levels, cortical thickness, and executive function in MCI patients have not yet been elucidated. We investigated the relationships among GABA levels, cortical thickness, and executive function in MCI patients.

**Methods:**

In this study, a total of 36 MCI patients and 36 sex-, age-, and education-matched healthy controls (HC) were recruited. But 33 MCI patients and 35 HC were included because of head motion or poor data quality for three MCI patients and one HC. The levels of gamma-aminobutyric acid plus relative to creatine (GABA+/Cr) and glutamate-glutamine relative to creatine (Glx/Cr) in the anterior cingulate cortex (ACC) and posterior cingulate cortex (PCC) were measured using the Meshcher-Garwood point resolved spectroscopy (MEGA-PRESS) sequence. Metabolite ratios, cortical thickness, and executive function and their interrelationships were determined in the MCI and HC groups.

**Results:**

Patients with MCI showed lower GABA+/Cr levels in the ACC and PCC. Combined levels of GABA+ and Glx in the ACC and GABA+ in the PCC showed good diagnostic efficacy for MCI (AUC: 0.82). But no differences in cortical thickness were found between the two groups. In the MCI group, lower GABA+/Cr level was correlated to worse performance on the digit span test backward, and the shape trail test-B. The cortical thickness was not associated with GABA+ levels and executive function in patients.

**Conclusion:**

These results implied that decreased GABA levels in the ACC and PCC had a critical role in the early diagnosis of impaired executive function of MCI. Therefore, GABA in the ACC and PCC could be a potential diagnostic marker of the executive function decline of MCI.

## 1. Introduction

Mild cognitive impairment (MCI) is an intermediate state between normal aging and dementia, characterized by the manifestation of mild cognitive symptoms along with alterations in the neurobiological function that may eventually result in neurodegeneration. The worldwide prevalence of MCI was over 15% among community dwellers aged 50 years and older (Albert et al., 2011; Bai et al., 2022). Epidemiological studies from China showed that the overall prevalence of dementia was 6.0%, and the MCI was 15.5% in adults over 60 years (Jia et al., 2020). In a previous study, MCI progressed to dementia in one-third of the people observed for 3–5 years (Manly et al., 2008). In order to offer appropriate support and prevent the quality of life from declining for MCI patients, diagnosis and intervention at the early stage are necessary.

MCI patients are commonly evaluated using neuropsychological scales, cerebrospinal fluid (CSF) biomarkers, and imaging methods, such as positron emission tomography (PET) and magnetic resonance imaging (MRI) (Ottoy et al., 2019; Lauretani et al., 2020; Santangelo et al., 2020). The evaluation of MCI patients using neuropsychological scales is crucial, as it involves assessing overall cognitive function and specific cognitive domains. Among these, executive function impairment leads to a slight but noticeable cognitive deficit in MCI besides memory impairment (Kirova et al., 2015; Junquera et al., 2020). The CSF and positron emission tomography (PET) biomarkers are considered critical not only for the diagnosis of MCI but also for the prediction of possible dementia progression (Albert et al., 2011). Research demonstrated that MCI patients exhibited decreased levels of Aβ1–42 in CSF, while PET showed reduced cerebral metabolism on [18F] fludeoxyglucose (FDG) PET and increased amyloid deposition on amyloid PET (Giau et al., 2019; Spallazzi et al., 2019). A study demonstrated that the CSF p-tau/Aβ (42) ratio and brain FDG-PET may reliably detect MCI “imminent” converters to AD (Santangelo et al., 2020). In addition, several MRI imaging studies found volumetric atrophy in MCI, such as the hippocampus, medial temporal lobe, etc. (Lehmann et al., 2013; Mak et al., 2017). However, the frequent clinical use of CSF and PET is limited due to the invasiveness of the procedures, cost, and radiation exposure. The MRI volume atrophy measurement may be insensitive to some MCI patients (Delli Pizzi et al., 2019). Therefore, it is necessary to explore the other markers for MCI patients.

The levels of neurotransmitters and the cortical thickness can reflect cognitive function. Thinning of the cortex in specific brain regions is associated with cognitive dysfunction dominated by that brain region (Ren et al., 2022). Gamma (γ)-aminobutyric acid (GABA) and glutamate (Glu) are important inhibitory and excitatory neurotransmitters in the central nervous system (CNS) and their abnormal levels can contribute to cognitive impairment (Bi et al., 2020). The known pathophysiology of MCI/AD includes amyloid β plaques (Aβ) deposition on several brain regions, including the anterior cingulate cortex (ACC) and posterior cingulate cortex (PCC) (Small et al., 2006; Wu et al., 2021) leading to cognitive deficits and neuropsychiatric symptoms (Hirao et al., 2015). However, inflammation, another pathogenesis in MCI/AD, may contribute to the disruption of GABA and Glu levels (Bradburn et al., 2019; Bi et al., 2020; Sood et al., 2021). Previous studies demonstrated that impaired GABAergic inhibitory networks (Long and Holtzman, 2019) and glutamate toxicity (Zott and Konnerth, 2023) played a role in the pathogenesis of AD. Thus, besides cortical thickness, the levels of these two neurotransmitters might identify MCI and subsequent cognitive progression. Furthermore, the stability of executive function is influenced by GABAergic processes (Hatoum et al., 2023). Brain regions relevant to executive function included the prefrontal cortex [mainly dorsolateral prefrontal cortex (DLPFC)] and the ACC, where the changes in GABA levels determined using magnetic resonance spectroscopy (MRS) (Li et al., 2022a) and cortical thinning determined using structural MRI (Makris et al., 2007) were reported. And the PCC is a crucial region of the default mode network, which was closely related to executive function (Brown et al., 2019). However, most of the existing studies have only focused on the correlation between GABA levels or cortical thickness and memory function in AD or MCI. The relationships among GABA levels, cortical thickness, and executive function in MCI have not yet been elucidated.

A molecular quantification technique, proton-MRS (^1^H-MRS) is available to measure the levels of endogenous brain neurochemicals (Mitolo et al., 2019). The GABA level in the brain is extremely low and the GABA level peaks by the traditional ^1^H-MRS overlap with other metabolites, such as N-acetyl aspartic acid (NAA), Glu, and glutamine (Gln) (Weis et al., 2021). Therefore, the Meshcher-Garwood point resolved spectroscopy (MEGA-PRESS) sequence (Mescher et al., 1998), using J-difference spectrum editing technology, was introduced to provide relatively rapid and reliable quantification of the CNS GABA concentration. But the detected signal was referred to as GABA plus (GABA+) instead of GABA, as it included contributions from both macromolecules (MM) as well as smaller homocysteine molecules. Additionally, this sequence could effectively include the glutamate-glutamine (Glx) signal due to the significant structural and chemical similarities between Glx and GABA (Harris et al., 2017). Further, 3D high-resolution T1-weighted sequence is mainly used to observe anatomical structures (Alhazmi et al., 2020), which in combination with advanced post-processing technique, such as FreeSurfer, a well-documented semi-automatic software for the cortical surface reconstruction and cortical thickness calculation (Fischl and Dale, 2000), allows for accurate determination of cortical thickness.

Combining molecular and structural imaging is a potential step for distinguishing MCI patients and evaluating their executive function impairment. The present study aimed to (i) compare the differences in GABA levels and cortical thickness in the ACC and PCC and executive function between the MCI patients and healthy older adults; (ii) assess the sensitivity and specificity for the diagnosis of MCI by utilizing combined neurometabolic content; (iii) explore the relationships among GABA levels, cortical thickness, and executive function in both groups.

## 2. Materials and methods

### 2.1. Participants

Thirty-six patients with MCI and 36 healthy controls (HC) were recruited from the neurology clinics of Union Hospital, Tongji Medical College, Huazhong University of Science and Technology, and underwent neuropsychological assessment and MRI scanning. But one participant with MCI was excluded due to motion artifacts during the scanning process, and two MCI patients and one HC were excluded based on post-processing spectral fitting criteria. Therefore, the study included 33 MCI patients and 35 HC. All participants enrolled in this study were (a) between 50 and 80 (included) years of age; (b) were right-handed; (c) had no major psychiatric disease (e.g., severe major depression, severe anxiety, bipolar disorder, and substance abuse); (d) had no active cancer; and (e) had no traumatic injuries, serious cardiovascular diseases, or hydrocephalus according to medical history and MRI images of all participants. Besides, the inclusion criteria for patients with MCI were as follows; (a) MCI reported by patients or insiders, or diagnosed by an experienced clinician; (b) Patients with cognitive dysfunction confirmed by objective tests [Montreal cognitive assessment, MoCA score of ≤24; ≤19 for ≥7; 1–6 years of education (Nasreddine et al., 2005; Lu et al., 2011), clinical dementia rating (CDR) of 0.5]; (c) Patients with preservation of independence in functional abilities; and (d) Patients who were not demented. The inclusion criteria for the HC group were as follows. Age-, sex-, and education-matched subjects to MCI group with normal cognitive function (MoCA score of ≥25; ≥20 for ≥7; 1–6 years of education; CDR of 0). Participants for both groups were excluded if they had (a) cognitive impairment due to other etiologies (Parkinson’s disease, Huntington’s disease, stroke, severe thyroid diseases, and metabolic diseases) and (b) contraindications to MRI scanning. Before the onset of the study, approval from the Institutional Ethics Committee and informed consent from each subject were obtained.

### 2.2. Neuropsychological assessment

All participants underwent neuropsychological tests which were conducted by an experienced neuropsychologist. The MoCA was used for global cognitive screening (Yu et al., 2012; Pinto et al., 2019), including for orientation, attention, memory, visuospatial/executive function, and naming function. Individual episodic memory performance, including immediate recall and delayed recall, was measured by the auditory verbal learning test (AVLT) (Zhao et al., 2012; Xu et al., 2020). The digit span test (Chincotta and Chincotta, 1996) consisted of a digit forward (FDS) assessment of attention and a backward (BDS) assessment of executive function. The shape trail test (STT) is a new version of the traditional trail-making test, in which attention is tested by the STT-A while the executive function is tested by the STT-B (Zhao et al., 2013). The Hamilton depression rating scale (HAMD) and the Hamilton anxiety rating scale (HAMA) were implemented to assess the emotional state. Participants were excluded if they had HAMD of ≥24 (Zimmerman et al., 2013) or HAMA of ≥29 that were considered severe depression and anxiety.

### 2.3. Neuroimaging

All subjects were scanned on a 3.0 T scanner (Philips Achieva TX; Philips, Best, Netherlands) using a 32-channel phased-array head coil. 3D high-resolution T1-weighted images were acquired using a magnetization-prepared turbo field echo sequence using the following parameters, repetition time/echo time (TR/TE) of 5.8/2.7 ms, flip angle of 8°, the field of view of 220× 220 × 180 mm^3^, slice thickness of 1 mm, and in-plane resolution of 1 × 1 mm^2^.

**Figure 1:**
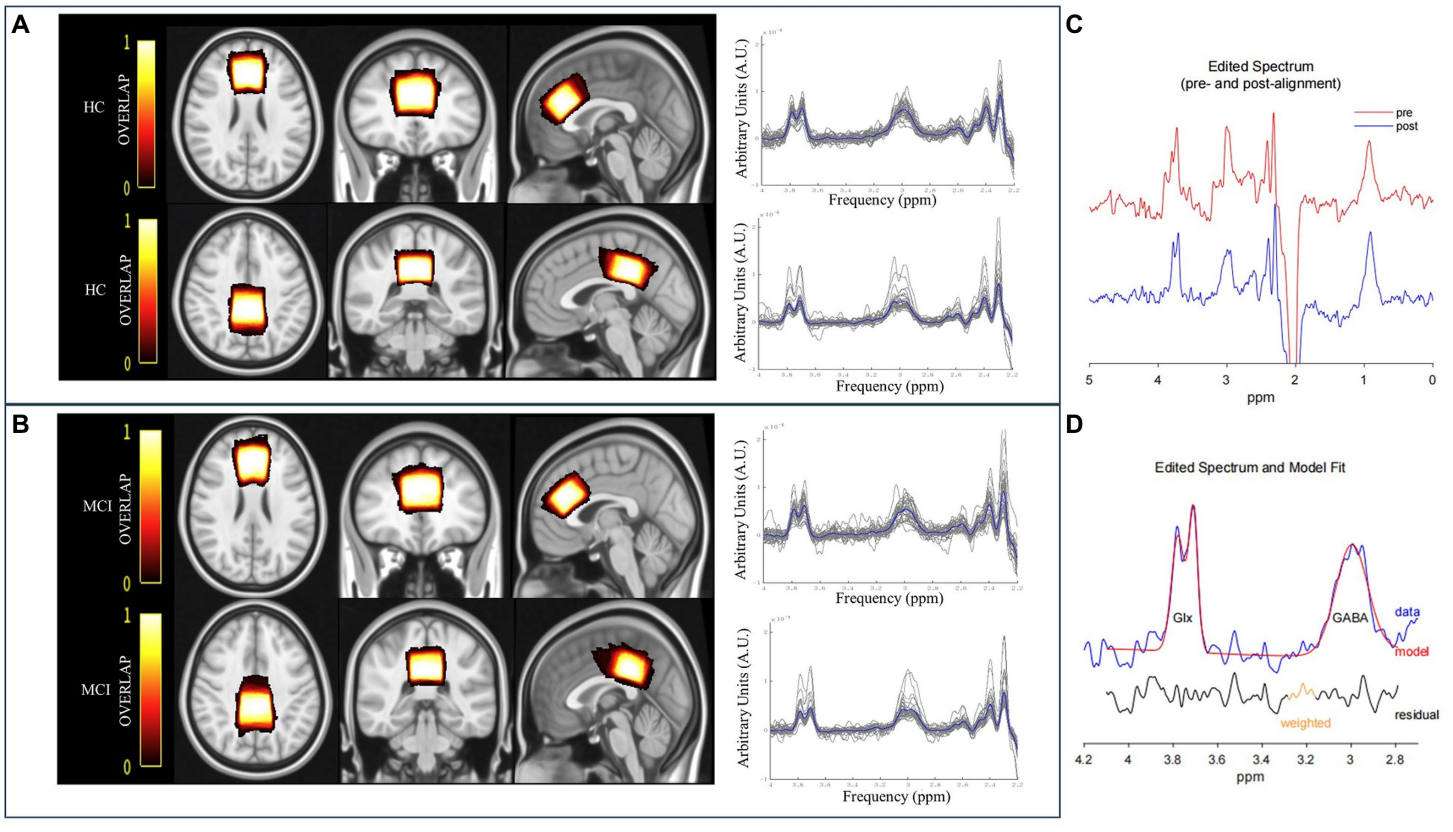
The overlay of the MRS voxel positions and raw spectra [individual (grey) and group mean (blue)] for HC **(A)** and MCI patients **(B)**. The color bar indicates the overlap of the individual voxels, with bright indicating a high overlap and dark color a low overlap. **(C)** GannetLoad output of the edited spectra. The red line indicates preprocessed spectrum before frequency and phase correction, and the blue line denotes the post-processed spectrum after frequency and phase correction. **(D)** GannetFit output of fitted GABA+ and Glx signals. The blue line represents the experimental data, the red line denotes the fit model, and the black line indicates the residual between the experimental data and the curve-fitted data.

Concentrations of GABA+, Glx to creatine (Cr) were obtained using the MEGA-PRESS sequence. A frequency-selective, refocusing Gauss pulse resonating at 1.89 ppm (the ON spectrum) and 7.46 ppm (the OFF spectrum) during odd and even-numbered acquisitions were applied, respectively. The brain volume of interest (VOI) was in the ACC (volume size = 30 × 30 × 20 mm^3^) and the PCC (volume size = 30 × 30 × 20 mm^3^) (Figures 1A,B) based on 3D high-resolution T1-weighted images, avoiding the lateral ventricle and skull. The median sagittal plane was selected as the reference plane for voxel localization. The voxels of the ACC and PCC were, respectively, positioned superior to the genu of the corpus callosum and posterior and superior to the splenium of the corpus callosum, both of which were aligned with the shape of the corpus callosum and positioned medial to the axial plane. The parameters of this sequence were as follows, TR/TE of 2000/68 ms; averages of 160 (consisting of 80 ON and 80 OFF resonance pulses); spectral bandwidth of 2000 Hz; acquisition time of 5:40 min per voxel (total scanning time of 11:20 min). Water suppression was conducted using the variable power and optimized relaxations delays (VAPOR) method. The checklist for MRS protocol and data quality control in Supplementary Table S1.

### 2.4. Data postprocessing

#### 2.4.1. MRS processing

The quantification of metabolite signals was assessed using a MATLAB-based GABA analysis toolkit, Gannet3.1.^1^ Subsequently, GABA+ and Glx signals were obtained at 3.02 ppm and 3.74 ppm, from the subtracted spectrum image of (ON–OFF) because the majority of the signals in the spectra were undisturbed by the editing pulse (Mullins et al., 2014) (Figures 1C,D). Meanwhile, individual and group mean of raw spectra were shown in Figures 1A,B. Metabolite ratios that were investigated were primarily referenced to Cr, obtained at 3.0 ppm from the OFF spectrum because it was more stable and robust than water referencing (Valenzuela and Sachdev, 2001; Bogner et al., 2010; Graff-Radford and Kantarci, 2013). The signal detected by the MEGA-PRESS at 3.02 ppm was a combination of contributions from GABA, macromolecules, and homocarnosine. Therefore, it was referred to as “GABA+” instead of GABA.

Head motion was checked when artifacts were present in the spectra, such as line splitting, or if there was signal from outside the organ of interest (e.g., subcutaneous lipid signals for brain MRS) (Andronesi et al., 2021). And we used (a) head immobilization; (b) prospective correction; and (c) retrospective correction to correct head motion during MRS acquisition for data analysis. The specific methods were included in Supplementary Table S1.

Data preprocessing included the automated frequency and phase correction, artifact rejection based on frequency deviation (>3 SD from the mean), and 3 Hz exponential line broadening. Metabolite concentrations were estimated by fitting Gaussian curves to the 3.02 ppm (GABA+) and double Gaussian curves to the 3.74 ppm (Glx) peaks and scaling them relative to creatine. Concentrations for GABA+ and Glx were reported in institutional units (i.u.). We kept the full width at half maximum (FWHM) value smaller than 20 Hz in order to control the quality of the MRS data. Meanwhile, individual metabolite ratios with a relative FitError above 15% were excluded (Liu et al., 2020).

#### 2.4.2. Volumes of interest position and segmentation

The overlapping individual spectra and an average voxel localization for both voxels, after transformation to Montreal Neurological Institute (MNI) space FSL software (FMRIB SOFTWARE LIBRARY, Oxford UK, FSL version 6.0) were shown in Figures 1A,B. Bright represented greater overlap between participants, and dark represented less overlap.

**Figure 2:**
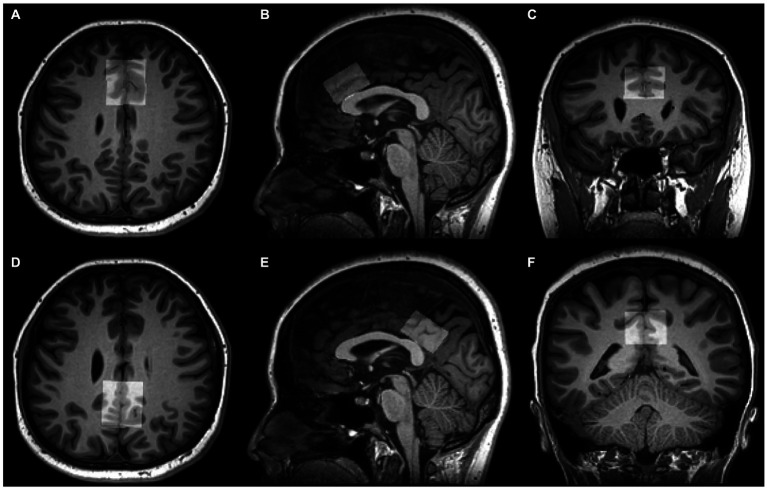
Voxel positions of tissue segmentation using SPM12. 3D T1-weighted images show the position and size of VOI in the ACC **(A–C)** and PCC **(D–F)** on axial, sagittal, and coronal planes.

The group differences in metabolite levels were modulated by the proportions of white matter (WM), gray matter (GM), and cerebrospinal fluid (CSF) in volumes of interest (VOIs). Therefore, preprocessing of 3D high-resolution T1-weighted images format conversion was first performed (from DICOM to NIfTI using MRIcroGL, https://www.nitrc.org/projects/mricrogl/), after which the SPM12 was applied to measure the GM, WM, and CSF volumes within MRS voxels (Figure 2).^2^ The GM, WM, and CSF fractions were obtained, and the ratio of GM/ (GM + WM) of each voxel was calculated.

#### 2.4.3. Cortical thickness measurement

**Figure 3:**
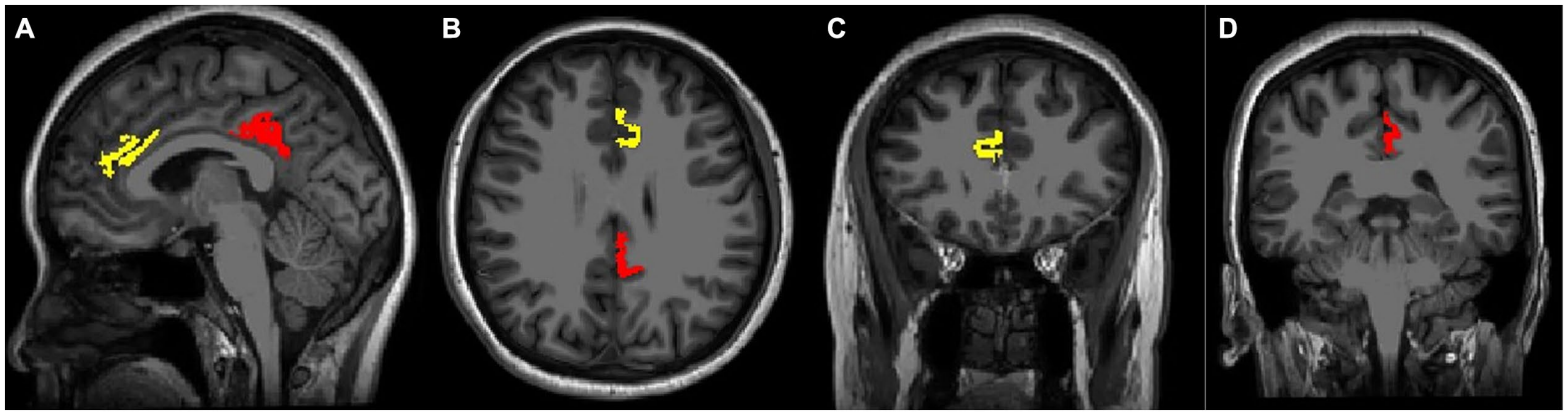
Voxel positions using Bainnetome Atlas. 3D T1-weighted images show the position of left cortical thickness in sagittal **(A)**, axial **(B)**, and coronal images **(C,D)**. The yellow mark represents the ACC thickness, the red color indicates the PCC thickness.

All 3D high-resolution T1-weighted images were automatically segmented using FreeSurfer version 7.2.0.^3^ All subjects were processed using the command recon-all default parameters. The measurement process involved several steps, including transformation to Talairach and MNI coordinate systems, removal of non-brain tissue, and segmentation of subcortical white and deep grey matter (Fischl et al., 2002; Ségonne et al., 2004). After intense normalization, the boundary between white and gray matter was separated and the topological defects were automatically corrected (Fischl et al., 2001). The grey/white matter pial boundary was identified as the greatest intense shift (Fischl et al., 2001), and any segmentation inaccuracies were manually edited. The resulting surface models were registered in the Bainnetome Atlas,^4^ and the cortical thickness was determined by averaging the shortest distance between a vertex on the pial surface and the grey/white matter boundary (Clarkson et al., 2011). In the present study, the cortex thickness of the A32p_l and A23d_l was extracted as the left ACC and left PCC thickness, similarly, A32p_r and A23d_r as the right ACC and right PCC thickness in all participants (Figure 3).

### 2.5. Statistical analysis

All statistical analyses were performed using SPSS software (version 25.0; SPSS Inc., Chicago, IL, United States). The normal distributions of demographic and neurocognitive scores, spectral data, segmentation results, and cortical thickness were verified by the Shapiro–Wilk test. Differences between the groups were assessed by the Mann–Whitney U test and independent two-sample t-test. A χ2 test was used to analyze the count data. A general linear model was applied to determine the group differences in metabolite ratios, cortical thicknesses, and neurocognitive scores, with age and gender, and education in years as covariates. The paired test was used to compare the distribution of metabolite levels of the ACC and PCC, as well as cortical thickness of different hemispheres within groups. Partial correlation analyses were performed to detect correlations of metabolites, cortical thickness in regions of interest, and executive function, adjusting for age, gender, and education in years. The receiver operating characteristic (ROC) analysis was used to evaluate the diagnostic efficacy of MCI using combined neurometabolic content, with significant difference of metabolite between groups as independent variables, age, gender, and education as covariates. A *p*-value of <0.05 was considered statistically significant.

## 3. Results

### 3.1. Demographic and neuropsychological characteristics of participants

Demographic data and neurocognitive results of subjects are summarized in Table 1. No significant differences in age (*p* = 0.153), gender (*p* = 0.959), education (*p* = 0.139), HAMA (*p* = 0.179), and HAMD (*p* = 0.407) were found between groups. MoCA score in the MCI group was significantly lower than in the HC group (*p* < 0.001). MCI patients performed worse on AVLT (including AVLT-immediate recall and AVLT-delayed recall; all *p*-values were <0.001), FDS (*p* = 0.007), BDS (*p* = 0.038), STT-A (*p* = 0.005), and STT-B (*p*<0.001), relative to the HC.

**Table 1:** Demographic, clinical, and neuropsychological characteristics of participants.

	MCI (*n* = 33)	HC (*n* = 35)	*p*-value
Age (years)	64.73 ± 8.74	62.12 ± 5.95	0.153
Gender (F/M)	13/20	14/21	0.959
BMI (kg/m^2^)	23.13 ± 2.93	23.52 ± 2.89	0.585
Edu (years)	10.55 ± 3.36	11.71 ± 3.06	0.139
HAMA	3 (2–6)	2 (0–5)	0.179
HAMD	1 (0–3)	2 (0–4)	0.407
Global cognition			
MoCA	20.48 ± 1.79	26.43 ± 2.33	<0.001***
Episodic memory			
AVLT-immediate recall^a^	13.12 ± 2.47	17.54 ± 4.43	<0.001***
AVLT-delayed recall^a^	5.97 ± 2.60	12.03 ± 3.88	<0.001***
Attention			
FDS^a^	6.76 ± 1.60	7.91 ± 1.07	0.007**
STT-A^a^	70.55 ± 23.62	53.63 ± 15.36	0.005**
Executive function			
STT-B^a^	179.21 ± 32.66	140.00 ± 33.35	<0.001***
BDS^a^	4.15 ± 1.35	4.86 ± 1.03	0.038*

MCI, mild cognitive impairment; HC, healthy control; HAMA, hamilton anxiety scale; HAMD, Hamilton depression scale; MoCA, montreal cognitive assessment; AVLT, auditory verbal learning test; BDS, digit span test backward; FDS, digit span test forward; STT, shape trails test. Data are presented as mean ± SD, number (percentage), or median (IQR-interquartile range). ^a^Represents corrected for the year, gender, and educational level. *p < 0.05, ** *p* < 0.01, ***p < 0.001.

### 3.2. Comparisons of metabolite concentrations

Metabolite data for all participants are shown in Table 2 and Supplementary Figure S1. After adjusting for age, gender, and education, significant group differences were found in the levels of GABA+/Cr in the VOIs. Compared to the HC group, patients with MCI had a significant decrease in GABA+/Cr ratios in the ACC (*p* = 0.001) and PCC (*p* = 0.009), and Glx/Cr ratios in the ACC (*p* = 0.046). However, no significant group difference was found in Glx/Cr levels in the PCC (*p* = 0.542). The GABA+/Cr levels in the ACC were significantly lower compared to the PCC in each group (MCI, *t* = −7.69, *p*<0.001; HC, *t* = −7.92, *p*<0.001), but no significant difference in Glx/Cr levels.

**Table 2:** Results of MRS analysis and tissue segmentation in measured voxels.

	MCI (*n* = 33)	HC (*n* = 35)	*p*-value
ACC			
GABA+/Cr^a^	0.09 ± 0.02	0.10 ± 0.01	0.001**
Glx/Cr^a^	0.07 ± 0.01	0.07 ± 0.01	0.046*
GABA+ fitting errors (%)	9.41 ± 3.10	8.30 ± 2.42	0.102
Glx fitting errors (%)	8.26 ± 3.02	7.82 ± 2.34	0.506
FWHM (Hz)	8.99 ± 0.68	8.97 ± 0.41	0.833
GM/(GM + WM) (%)	55.67 ± 5.35	55.57 ± 4.31	0.936
PCC			
GABA+/Cr^a^	0.11 ± 0.01	0.12 ± 0.01	0.009**
Glx/Cr^a^	0.07 ± 0.01	0.07 ± 0.01	0.542
GABA+ fitting errors (%)	7.33 ± 1.90	7.15 ± 1.70	0.669
Glx fitting errors (%)	7.77 ± 2.30	8.03 ± 2.49	0.649
FWHM (Hz)	8.99 ± 0.68	8.97 ± 0.41	0.925
GM/(GM + WM) (%)	59.73 ± 3.78	59.51 ± 3.54	0.811

MCI, mild cognitive impairment; HC, healthy control; GABA+, GABA plus co-edited macromolecules and homocarnosine; Glx, glutamate-glutamine; FWHM, full-width at half-maximum; GM, gray matter; WM, white matter. Data are presented as mean ± SD. ^a^Represents corrected for the year, gender, and educational level. *p < 0.05, ** *p* < 0.01, ***p < 0.001.

### 3.3. The VOIs segmental results and MRS quality

The average GM tissue fraction GM/(GM + WM) was 55.67% and 59.73% in the ACC and PCC, respectively, in MCI patients, while in HC, it was 55.57% and 59.51%, respectively (Table 2). No significant differences in GM/(GM + WM) in the VOIs within groups (ACC, *p* = 0.936; PCC, *p* = 0.811). The fitting errors of metabolites and the FWHM in both VOIs did not differ between patients and HC (all *p*-values were > 0.05) (Table 2).

### 3.4. Accuracy of metabolite concentrations in diagnosing MCI

**Figure 4:**
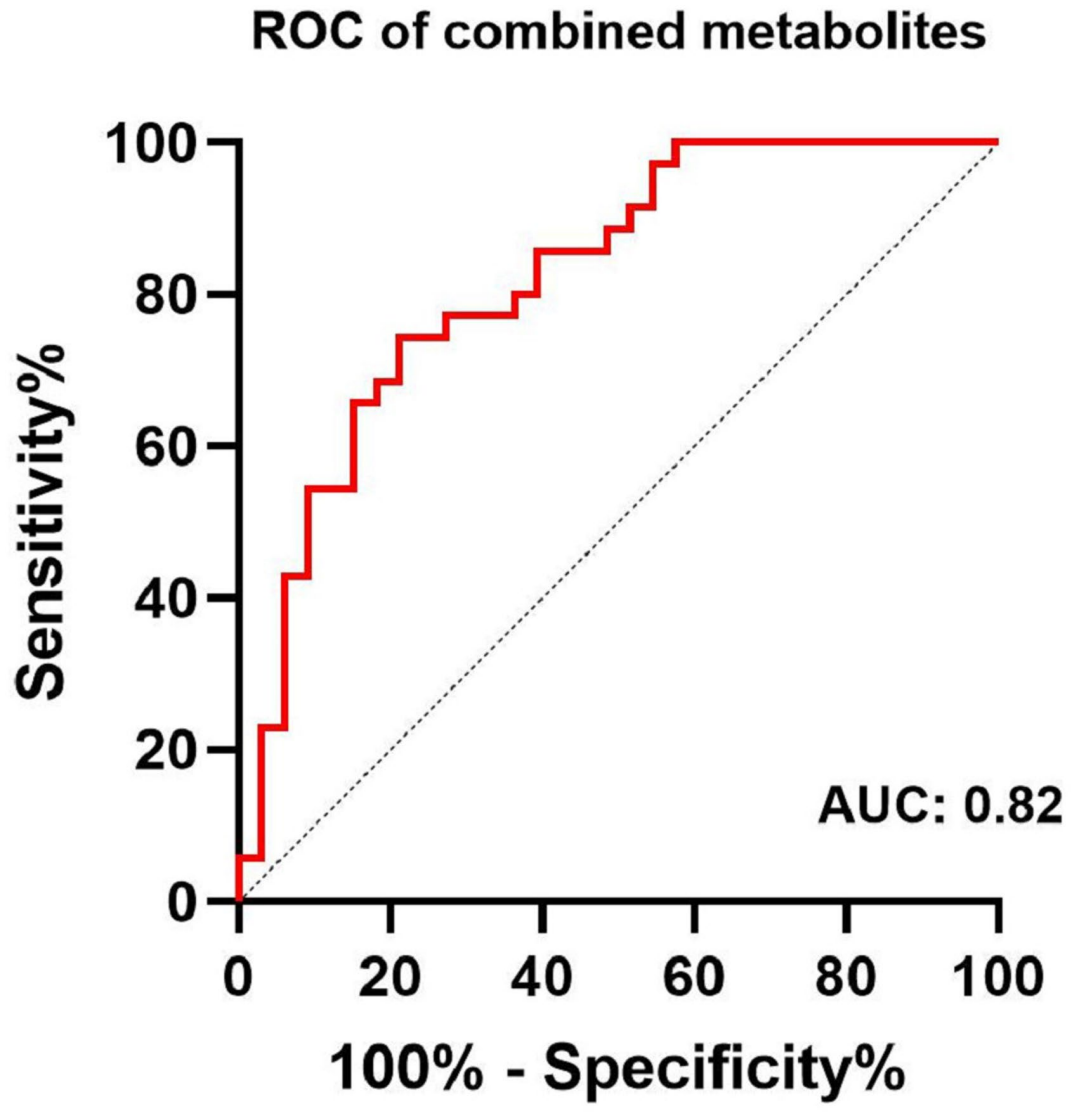
Receiver-operator-characteristic (ROC) analysis of combined content of GABA+ and Glx in the ACC, and GABA+ in the PCC for the diagnosis of MCI.

The largest area under the ROC curve for the combined content of GABA+ and Glx in the ACC and GABA+ in the PCC was 0.82 (95% confidence interval, 0.72–0.92, and with 78.8% sensitivity and 74.3% specificity), with age, gender, and education as covariates (Figure 4).

### 3.5. Comparison of cortical thickness

No significant differences in bilateral ACC and PCC thickness were found between groups (all *p*-value were >0.05) (Supplementary Table S2, Supplementary Figure S2). The right ACC thickness was thicker than left ACC thickness within groups (MCI, *t* = −2.90, *p*<0.01; HC, *t* = −5.40, *p*<0.001), while no significant difference was observed in the PCC.

### 3.6. Correlations among neuro metabolites, cortical thickness, and executive function

#### 3.6.1. Correlation between neuro metabolites and executive function

**Figure 5:**
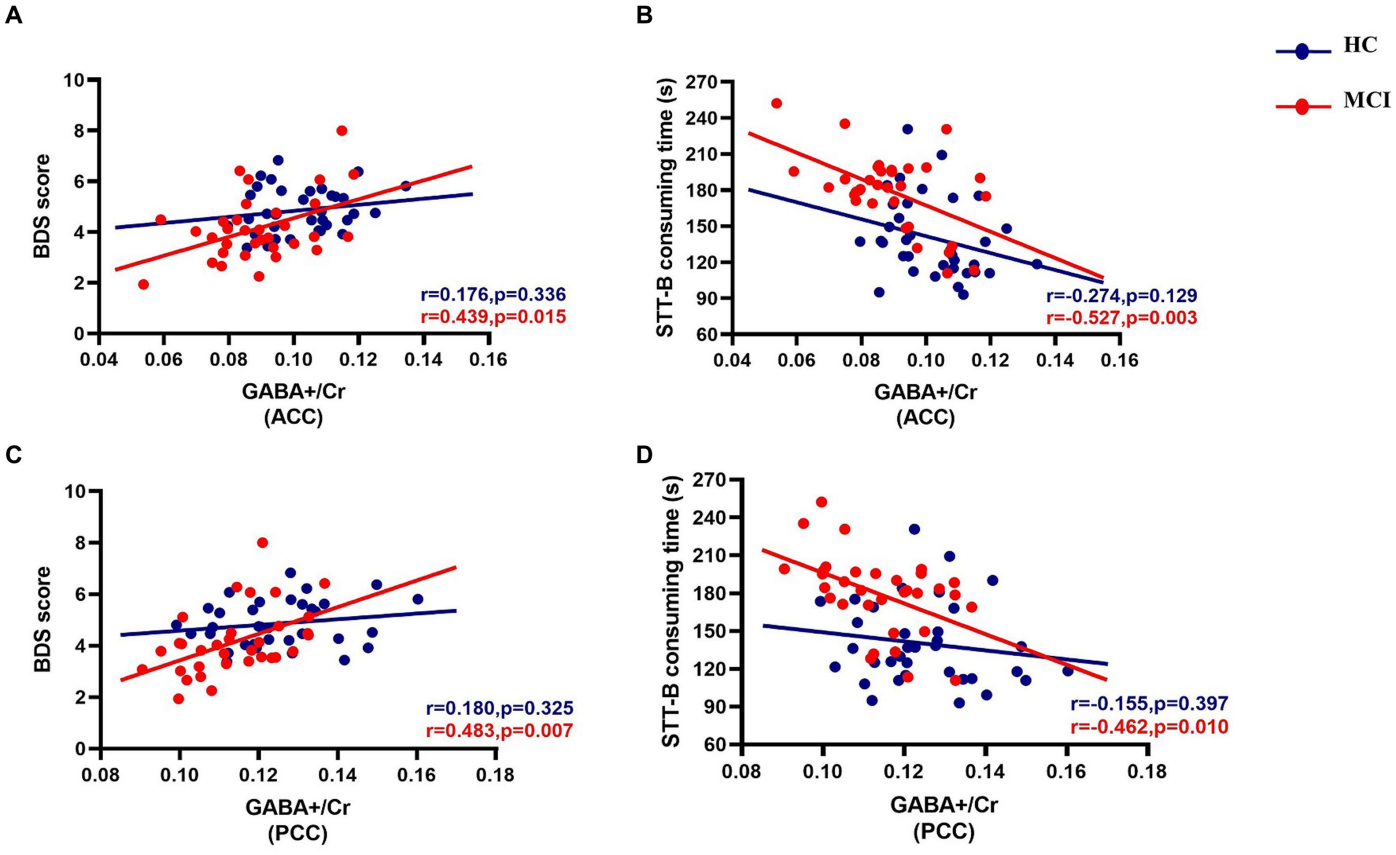
Partial correlations between GABA+/Cr levels of the ACC and PCC and executive function in both groups, adjusting for age, gender, and education (in years). The MCI group show statistically significant positive correlations between GABA+/Cr and BDS [ACC, *r* = 0.439, *p* = 0.015 **(A)**; PCC, *r* = 0.483, *p* = 0.007 **(C)**] and negative correlations between GABA+/Cr and STT-B [ACC, *r* = −0.527, *p* = 0.003 **(B)**; PCC, *r* = −0.462, *p* = 0.010 **(D)**] consuming time.

Correlations between GABA+ in both VOIs and executive function are summarized in Figure 5. In the MCI group, significant positive correlations between GABA+/Cr and BDS (ACC, *r* = 0.439, *p* = 0.015; PCC, *r* = 0.483, *p* = 0.007), and negative correlations between GABA+/Cr and STT-B (ACC, *r* = −0.527, *p* = 0.003; PCC, *r* = −0.462, *p* = 0.010), adjusting for age, gender, and education, were found. The relationship between GABA+/Cr ratios in the two regions and executive function did not reach statistical significance (all *p* values were > 0.05) in the HC group. Meanwhile, we found no relationships between Glx/Cr ratios in the VOIs and executive function within both groups (all *p* values were > 0.05).

#### 3.6.2. Correlation between cortical thickness and executive function

In the MCI group, no correlations were found between cortical thickness in VOIs and executive function, with age, gender, and education as covariates (all *p* values were > 0.05) (Supplementary Table S3). However, in the HC group, no correlation was found between cortical thickness and executive function, except for a positive correlation between the right PCC thickness and STT-B (*r* = 0.369, *p* = 0.038) (Supplementary Table S4).

#### 3.6.3. Correlation between neuro metabolites and cortical thickness

Within the ACC and PCC, the GABA+ and Glx levels were not significantly related to cortical thickness in the MCI patients, adjusting for age, gender, and education (all *p*-values were > 0.05) (Supplementary Tables S5, S6). In the HC group, only the Glx content of the ACC was positively correlated with the thickness of the right ACC (*r* = 0.371, *p* = 0.036) (Supplementary Tables S7, S8).

## 4. Discussion

This study found decreased GABA+ levels in the ACC and PCC and decreased Glx levels in the ACC in the MCI group. Combined levels of GABA+ and Glx in the ACC and GABA+ in the PCC showed good diagnostic efficacy for MCI (AUC: 0.82). Further, executive function was positively related to the GABA+ concentrations in the ACC and PCC within MCI patients. In contrast, cortical thickness could not distinguish MCI and healthy adults, and it was not related to the GABA level as well as executive function. Therefore, decreased GABA levels in the ACC and PCC had an important role in the early recognition of impaired executive function in the MCI patients, but not the cortical thickness.

GABA+ and Glx levels in the ACC were decreased in patients with MCI. MCI is the transition state from normal cognition to dementia, characterized by single or multiple cognitive dysfunctions but basic activities of daily living being preserved (Lissek and Suchan, 2021). About one-third of MCI people progress into irreversible dementia within 3–5 years (Manly et al., 2008), therefore, early and timely intervention at this stage provides significant benefits. An imbalance of neurotransmitters (such as GABA and Glu or Gln) in the brain of MCI or dementia patients exists and this may be due to inflammatory responses involving astrocytes. For example, neuroimaging studies found decreased GABA, Glx or Glu levels in the MCI (Riese et al., 2015; Oeltzschner et al., 2019) and AD patients (Lanctôt et al., 2004; Bai et al., 2015), although the affected brain regions were different. This study found that decreased GABA+ levels in the ACC and PCC and Glx levels in the ACC in the MCI group compared to the HC group were consistent with the abovementioned studies. Contrastingly, another study (Huang et al., 2017) found no significant difference in the GABA+/Cr and Glx/Cr ratios of ACC and right hippocampus between the MCI group and the elderly normal, which was different from our results. The discrepancy may have been due to population heterogeneity, differences in location, and the size of selected brain regions.

The decreased GABA levels may also have been due to GABAergic neuronal loss or/and dysfunction because the single photon emission computed tomography (SPECT) demonstrated reduced GABA_A_/benzodiazepine (BZD) receptor density in the parietal lobe of patients with MCI (Pappata et al., 2010). Additionally, the excitatory and inhibitory interneurons use Gln, the precursor of Glu synthesized in astrocytes, to synthesize Glu and GABA, and the released neurotransmitters are cleared by astrocytes (Czapski and Strosznajder, 2021; Sood et al., 2021; Kilb and Kirischuk, 2022). Therefore, the decline in GABA+ and Glx found in this study could be due to loss of interneurons, decreased GABA/Glu synthesis, or altered astroglial cycling of GABA, Glu, and Gln. Interestingly, we found that the GABA levels in the ACC were lower compared to the PCC within groups. But this observation required further validation through large-scale studies. Meanwhile, the diagnostic efficacy of combining GABA+ and Glx levels in the ACC and GABA+ levels in the PCC was excellent, suggesting the potential role of neuro metabolites in diagnosing MCI.

Further, executive function was correlated to the GABA+ levels in the ACC and PCC in MCI patients. The cingulate cortex is an essential component of the default mode network, and the PCC performs a complex exchange of information with the ACC to control cognitive function and emotion (Bubb et al., 2018; Aponik-Gremillion et al., 2022). The executive function represents a series of advanced cognitive control abilities necessary for achieving goals (Li et al., 2022a). Its impairment is a small but noticeable cognitive deficit in MCI besides impaired memory. Most of the existing imaging studies on executive impairment focused on the prefrontal/cerebellar cortex or white matter (Friedman and Robbins, 2022; Li et al., 2022b; Gustavson et al., 2023), and there are a few studies on the correlation between neuro metabolites and executive function in the ACC and PCC. The relationship between the execution and GABA, which has been discussed previously, was controversial (Marenco et al., 2018; Oeltzschner et al., 2019). A genome-wide association study found that executive function was influenced by GABAergic processes (Hatoum et al., 2023). In another research (Marenco et al., 2018) with middle-aged healthy volunteers, higher GABA levels in dorsal ACC were associated with better executive function measured by the Wisconsin card sorting test. Our results were consistent with the abovementioned studies. A positive correlation between execution and GABA was not only found in the ACC but also in the PCC. However, a previous study (Gozdas et al., 2022) examined the association between proton MRS metabolites in the DLPFC and cognitive function in amnestic mild cognitive impairment patients (aMCI). They identified that Glx/total Cr (tCr) levels, rather than GABA/tCr, were significantly associated with executive function in patients with aMCI. In previous research (Oeltzschner et al., 2019), the GABA levels of the ACC and PCC in the aMCI group were not related to the executive function measured by the symbol digit modalities test (SDMT). Different methods of assessing executive function, brain regions, and sample size might have contributed to the heterogeneity of outcomes.

In this study, brain atrophy was explored using the MRI modality. A study (Zhu et al., 2021) showed the left ACC and left PCC in MCI patients with moderate or severe WMH became thinning. They also found the cortical thickness of the middle frontal gyrus mediated the association between WMH presence and worse processing speed and executive function. We found no significant thinning of the ACC and PCC between groups and no correlation between executive function and cortical thickness, although the right ACC thickness was thicker than the left ACC thickness within groups. These observations could be attributed to the absence of WMH in MCI patients of our study. These differences also indicated that the brain atrophy pattern probably differed between MCI with and without WMH. There was an initial observation of atrophy occurring in the medial temporal lobe, including the entorhinal cortex and hippocampus in MCI and AD patients (Chandra et al., 2019). Since this study only measured the ACC and PCC, other brain areas may be needed to be explored in the future. For the first time, we also did not observe any correlation between cortical thickness and GABA+ or Glx levels in the ACC and PCC. Consequently, measuring neurometabolites might offer additive value in reflecting the executive impairment of MCI patients, rather than the cortical thickness of the ACC and PCC.

In light of the notable advancements in the outcomes, this study had several limitations. First, the non-significant findings in this study might have resulted from the small sample size. Second, whether GABA level changed with the MCI progression was unclear. These two limitations could be addressed by conducting longitudinal studies with larger sample sizes. Additionally, the measured metabolites did not represent the pure GABA and Glu content because GABA+ was the mixture of GABA, macromolecular, and homocarnosine, and Glx was the complex of Glu and Gln, which might have introduced bias into the results. The isolated changes in homocarnosine are unlikely to be the main driver of the observed changes, as the concentration of homocarnosine *in vivo* is considerably lower than GABA (Govindaraju et al., 2000). Therefore, new methods for macromolecule suppression of GABA-edited MRS (Chan et al., 2021), detecting “pure GABA,” should be used in future studies of MCI. Finally, the inclusion of additional regions is important in future studies, because the single-voxel MRS was not able to capture the relationships among GABA levels, cortical thickness, and executive function in other brain regions in this study.

## 5. Conclusion

The associations among GABA levels, cortical thickness, and executive function were investigated in MCI patients. Lower levels of GABA in the ACC and PCC were significantly associated with worse executive function in MCI patients. The study also demonstrated that GABA levels were more sensitive than structural changes in the ACC and PCC as indicators of executive function decline in MCI patients. These results suggested that GABA levels in the ACC and PCC could serve as potential diagnostic markers of executive function impairment in MCI.

## Data availability statement

The raw data supporting the conclusions of this article will be made available by the authors, without undue reservation.

## Ethics statement

The studies involving humans were approved by the Ethics Committee of Union Hospital, Tongji Medical College, Huazhong University of Science and Technology. The studies were conducted in accordance with the local legislation and institutional requirements. The participants provided their written informed consent to participate in this study.

## Author contributions

XF: data curation, data analysis and interpretation, methodology, and writing original draft. MQ: conceptualization, writing original draft, writing-review, and editing. XL, LC, LZ, and XZ: data analysis and interpretation. YL and QZ: data curation. PS and LL: software, methodology, and statistical analysis. YS: supervision, study design, project administration, and funding acquisition. JW: supervision, study design, project administration, writing-review and editing, and funding acquisition. All authors contributed to the article and approved the submitted version.

## Funding

This work was supported by the Ministry of Science and Technology of the People’s Republic of China (STI2030-Major Projects 2021ZD0201900) and the National Science Foundation of Hubei Province (2021CFB447).

## Conflict of interest

The authors declare that the research was conducted in the absence of any commercial or financial relationships that could be construed as a potential conflict of interest.

## Publisher’s note

All claims expressed in this article are solely those of the authors and do not necessarily represent those of their affiliated organizations, or those of the publisher, the editors and the reviewers. Any product that may be evaluated in this article, or claim that may be made by its manufacturer, is not guaranteed or endorsed by the publisher.

## Footnotes

^1^https://github.com/richardedden/Gannet3.1

^2^http://www.fil.ion.ucl.ac.uk/spm/software/spm12/

^3^http://surfer.nmr.mgh.harvard.edu

^4^http://atlas.brainnetome.org/bnatlas.html
