# Supplementary material for: Decreased GABA levels of the anterior and posterior cingulate cortex are associated with executive dysfunction in mild cognitive impairment
Source: Front Neurosci. 2023 Aug 11;17:1220122. doi: 10.3389/fnins.2023.1220122 (PMC10450953; doi:10.3389/fnins.2023.1220122)
Supplement: Supplementary file 1 [file Data_Sheet_1.ZIP › Supplementary Material.docx]

**Table S1.** **The checklist for MRS protocol and data quality control**

| **1. Hardware** |  |
| --- | --- |
| a. Field strength [T] | 3.0 T |
| b. Manufacturer | Philips |
| c. Model (software version if available) | Achieva TX |
| d. RF coils: nuclei (transmit/receive), number of channels, type, body part | A 32-channel phased-array head coil |
| e. Additional hardware | N/A |
| **2. Acquisition** |  |
| a. Pulse sequence | Meshcher-Garwood point resolved spectroscopy (MEGA-PRESS) |
| b. Volume of interest (VOI) locations | Anterior cingulate cortex, posterior cingulate cortex |
| c. Nominal VOI size [mm^3^] | 30×30×20 mm^3^ |
| d. Repetition time (TR), echo time (TE) [ms] | TR=2000ms, TE =68 ms |
| e. Total number of excitations or acquisitions per spectrum | 10 increments with 16 averages per increment |
| f. Additional sequence parameters | The spectral bandwidth of 2000 Hz, 1024 points |
| g. Water suppression method | VAPOR |
| **3. Data analysis methods and outputs** |  |
| a. Analysis software | GANNET 3.1 toolkit based on MATLAB |
| b. Data preprocessing (including the GannetLoad and GannetFit modules) | The GannetLoad module parses certain variables from the data headers, corrects the frequency and phase of individual spectra;  The GannetFit module fits GABA+ and Glx signals and assesses their relative concentrations to creatine |
| c. Output measure | Ratio to creatine |
| **4. Motion correction** |  |
| a. head immobilization | Sponge blocks and noise-canceling headphones |
| b. prospective correction. | B0 shim correction |
| c. retrospective correction | Frequency and phase correction using Spectral Registration, artifact rejection based on frequency deviation (>3 SD from the mean), and 3 Hz exponential line broadening |
| **5. Data quality** |  |
| a. Data exclusion criteria | FWHM above 20 Hz |
| b.Quality measures of postprocessing model fitting | Poor-fitting performance of FitError above 15% |

**Table S2.** Cortical thickness of participants

|  | MCI | HC | p value^a^ |
| --- | --- | --- | --- |
| lh-ACC thickness | 2.40±0.27 | 2.33±0.27 | 0.171 |
| lh-PCC thickness | 2.77±0.16 | 2.78±0.17 | 0.993 |
| rh-ACC thickness | 2.56±0.32 | 2.61±0.27 | 0.896 |
| rh-PCC thickness | 2.78±0.14 | 2.81±0.14 | 0.853 |

^a^ represented corrected for year, gender and educational level. Abbreviations: MCI , mild cognitive impairment; HC, healthy control; lh, left hemisphere; rh, right hemisphere; ACC, anterior cingulate cortex; PCC, posterior cingulate cortex.

**Table S3.** Partial correlation coefficient between cortical thickness and executive function in MCI group

|  | lh-ACC thickness | | rh-ACC thickness | | lh-PCC thickness | | rh-PCC thickness | |
| --- | --- | --- | --- | --- | --- | --- | --- | --- |
|  | r | p value^a^ | r | p value^a^ | r | p value^a^ | r | p value^a^ |
| BDS | -0.082 | 0.667 | -0.128 | 0.500 | 0.087 | 0.646 | -0.041 | 0.831 |
| STT-B | -0.115 | 0.545 | -0.076 | 0.691 | -0.086 | 0.651 | 0.026 | 0.892 |

^a^ represented corrected for year, gender and educational level. Abbreviations: lh, left hemisphere; rh, right hemisphere; ACC, anterior cingulate cortex; PCC, posterior cingulate cortex; BDS, Digit span test backward; STT, Shape trail test.

**Table S4.** Partial correlation coefficient between cortical thickness and executive function in HC group

|  | lh-ACC thickness | | rh-ACC thickness | | lh-PCC thickness | | rh-PCC thickness | |
| --- | --- | --- | --- | --- | --- | --- | --- | --- |
|  | r | p value^a^ | r | p value^a^ | r | p value^a^ | r | p value^a^ |
| BDS | -0.007 | 0.969 | -0.002 | 0.993 | 0.156 | 0.394 | -0.006 | 0.975 |
| STT-B | 0.272 | 0.132 | 0.148 | 0.419 | 0.349 | 0.050 | 0.369 | 0.038* |

^a^ represented corrected for year, gender and educational level. Abbreviations: lh, left hemisphere; rh, right hemisphere; ACC, anterior cingulate cortex; PCC, posterior cingulate cortex; BDS, Digit span test backward; STT, Shape trail test.*p＜0.05.

**Table S5.** Partial correlation coefficient between neurometabolites and cortical thickness in the ACC in MCI group

|  | GABA+/Cr | | Glx/Cr | |
| --- | --- | --- | --- | --- |
|  | r | p value^a^ | r | p value^a^ |
| lh-ACC thickness | 0.289 | 0.121 | -0.244 | 0.194 |
| rh-ACC thickness | -0.170 | 0.368 | 0.053 | 0.782 |

^a^ represented corrected for year, gender and educational level. Abbreviations: GABA+, GABA plus co-edited macromolecules and homocarnosine; Glx, glutamate-glutamine; lh, left hemisphere; rh, right hemisphere; ACC, anterior cingulate cortex; PCC, posterior cingulate cortex.

**Table S6.** Partial correlation coefficient between neurometabolites and cortical thickness in the PCC in MCI group

|  | GABA+/Cr | | Glx/Cr | |
| --- | --- | --- | --- | --- |
|  | r | p value^a^ | r | p value^a^ |
| lh-PCC thickness | -0.211 | 0.262 | 0.183 | 0.333 |
| rh-PCC thickness | -0.304 | 0.102 | 0.138 | 0.467 |

^a^ represented corrected for year, gender and educational level. Abbreviations: GABA+, GABA plus co-edited macromolecules and homocarnosine; Glx, glutamate-glutamine; lh, left hemisphere; rh, right hemisphere; ACC, anterior cingulate cortex; PCC, posterior cingulate cortex.

**Table S7.** Partial correlation coefficient between neurometabolites and cortical thickness in the ACC in HC group

|  | GABA+/Cr | | Glx/Cr | |
| --- | --- | --- | --- | --- |
|  | r | p value^a^ | r | p value^a^ |
| lh-ACC thickness | -0.045 | 0.805 | 0.012 | 0.946 |
| rh-ACC thickness | -0.025 | 0.893 | 0.371 | 0.036* |

^a^ represented corrected for year, gender and educational level. Abbreviations: GABA+, GABA plus co-edited macromolecules and homocarnosine; Glx, glutamate-glutamine; lh, left hemisphere; rh, right hemisphere; ACC, anterior cingulate cortex; PCC, posterior cingulate cortex.*p＜0.05.

**Table S8.** Partial correlation coefficient between neurometabolites and cortical thickness in the PCC in HC group

|  | GABA+/Cr | | Glx/Cr | |
| --- | --- | --- | --- | --- |
|  | r | p value^a^ | r | p value^a^ |
| lh-PCC thickness | 0.267 | 0.139 | -0.029 | 0.873 |
| rh-PCC thickness | 0.172 | 0.348 | 0.075 | 0.683 |

^a^ represented corrected for year, gender and educational level. Abbreviations: GABA+, GABA plus co-edited macromolecules and homocarnosine; Glx, glutamate-glutamine; lh, left hemisphere; rh, right hemisphere; ACC, anterior cingulate cortex; PCC, posterior cingulate cortex.
