# Supplementary figures and images for: Decreased GABA levels of the anterior and posterior cingulate cortex are associated with executive dysfunction in mild cognitive impairment
Source: Front Neurosci. 2023 Aug 11;17:1220122. doi: 10.3389/fnins.2023.1220122 (PMC10450953; doi:10.3389/fnins.2023.1220122)

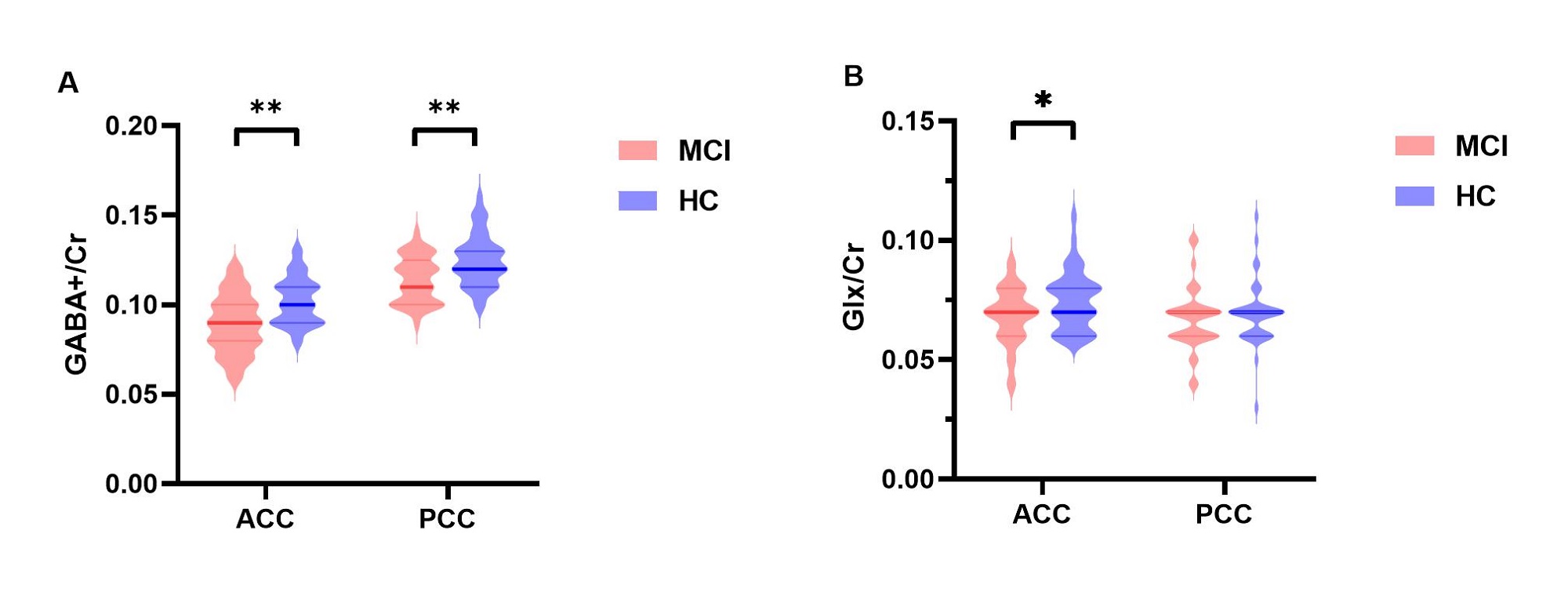

Supplement: Supplementary file 1 [file Data_Sheet_1.ZIP › Figure S1.jpg]

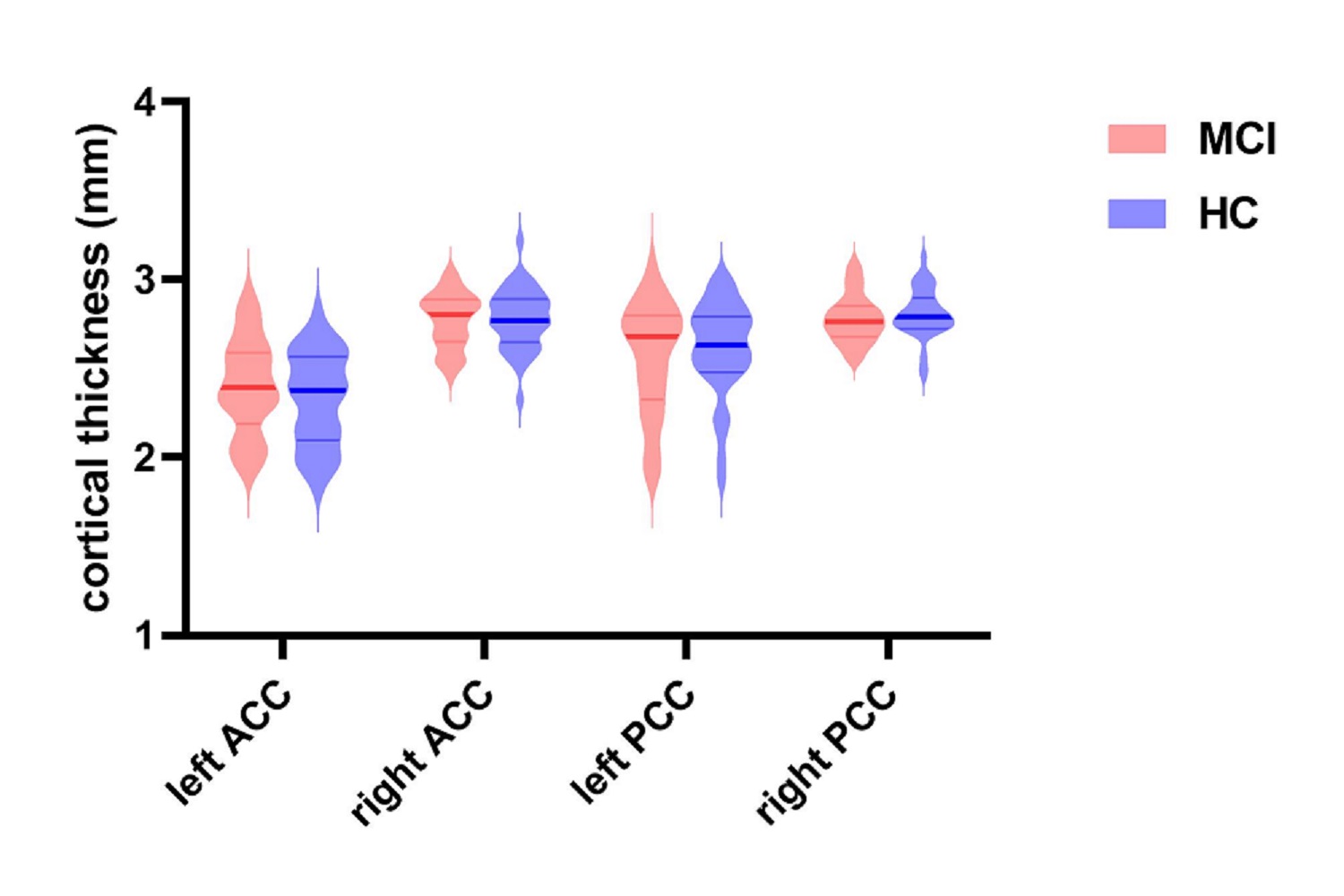

Supplement: Supplementary file 1 [file Data_Sheet_1.ZIP › Figure S2.jpg]
